# Revictimization of Violence Suffered by Those Diagnosed with Alcohol Dependence in the General Population

**DOI:** 10.1155/2015/805424

**Published:** 2015-04-27

**Authors:** F. G. Moreira, M. I. Quintana, W. Ribeiro, R. A. Bressan, M. F. Mello, J. J. Mari, S. B. Andreoli

**Affiliations:** ^1^Universidade Federal de São Paulo (UNIFESP), 04038-020 São Paulo, SP, Brazil; ^2^Universidade Católica de Santos (UNISANTOS), 11015-002 Santos, SP, Brazil

## Abstract

*Objective*. To verify the association between violence and alcohol dependence syndrome in sample populations.* Method*. Population-wide survey with multistage probabilistic sample. 3,744 individuals of both genders, aged from 15 to 75 years, were interviewed from the cities of São Paulo and Rio de Janeiro using the Composite International Diagnostic Interview (CIDI 2.1).* Results*. In both cities, alcohol dependence was associated with the male gender, having suffered violence related to criminality, and having suffered familial violence. In both cities, urban violence, in more than 50% of cases, and familial violence, in more than 90% of cases, preceded alcohol dependence. The reoccurrence of traumatic events occurred in more than half of individuals dependent on alcohol. In São Paulo, having been diagnosed with PTSD is associated with violence revictimization (*P* = 0.014; Odds = 3.33).* Conclusion*. Alcohol dependence syndrome is complexly related to urban and familial violence in the general population. Violence frequently precedes alcoholism, but this relationship is dependent on residence and traumatic events. This vicious cycle contributes to perpetuating the high rates of alcoholism and violence in the cities. Politicians ordering the reduction of violence in the large metropolises can, potentially, reduce alcoholism and contribute to the break of this cycle.

## 1. Introduction

Disorders related to alcohol are included in the ten most important issues in public health worldwide; alcohol dependence syndrome is the fifth most incapacitating disease in the Americas and Europe [1]. Within those factors that can be highlighted that make this disease so incapacitating is the relationship between alcoholism and the occurrence of accidents and violence [2].

The association between alcohol use and accidents occurs frequently, especially in traffic [3, 4]. Among individuals involved in traffic accidents, 61% tested positive for alcohol, of which 56% were run over and 70% were victims of shock and vehicle rollover [5]. In the city of Recife, during the popular party Carnival, 88% of victims who died in traffic accidents tested positive for alcohol [6].

On the other hand, having been a victim of a traumatic situation and the development of pathological use of alcohol have also been found to be related [7]. Experiencing violence during childhood [8] and witnessing violence during childhood [9] have been identified as precursors to alcoholism in adults. The relationship between alcohol use, violence, and diverse psychiatric disorders, including Posttraumatic Stress Disorder (PTSD), has been shown in population-wide studies [3].

Although it is clear that some traumatic events, such as accidents, are associated with a rise in the improper use of alcohol, the relationship between alcohol use and violent traumas is not so clear. The discussion of whether violent traumas precede or are triggers of alcohol dependence syndrome and predispose individuals to revictimization has been the focus of several studies [8, 10, 11]. In this sense, the focus of the present study was to investigate these relationships using data collected from the “Epidemiological Study of Violence and Post Traumatic Stress in the Cities of São Paulo and Rio de Janeiro” [12].

## 2. Methods

With 11,244,369 and 6,323,037 habitants, respectively, São Paulo and Rio de Janeiro are the two most populated metropolises in Brazil and are also among the most violent cities in the country [13]. In 2003, the average rate of homicides in these cities was 47.13 and 44.3 per 100,000 habitants, respectively, while the rate in the country was 28.6. Like the social indicators, the homicide rates vary considerably within the cities; for example, in 2003, the rates varied between 2.90 and 88.20 within the 96 administrative districts of São Paulo and between 0 and 91.77 within the 33 administrative regions of Rio de Janeiro [14–16].

Between June 2007 and July 2008, a cross-sectional, epidemiological study was conducted in one phase in these two cities, with the multistage probabilistic sample representative of the population aged 15 to 75 years. A company specialized in household surveys, the Brazilian Institute of Public Opinion and Statistics (IBOPE), was hired to carry out the fieldwork, under supervision of the authors, members of the CIDI Training Center WHO/Brazil/UNIFESP. The authors had open access to the team of raters engaged in the project and were responsible for the training. The training course comprised a 30-hour theoretical and practical module [12].

To select the sample, different areas of the cities were classified according to homicide rates and later divided into seven groups (1 = less than 10 homicides per 100,000 habitants; 2 = 10.01 to 20; 3 = 20.01 to 30; 4 = 30.01 to 40; 5 = 40.01 to 50; 6 = 50.01 to 60; 7 = more than 60 homicides per 100,000 habitants). Then, all of the census-based sectors within each group were mapped and randomly drawn. The number of census-based sectors within each group varied between 4 and 18, according to the size of the population in each group. In each census-based sector, 43 cases (São Paulo) and 30 cases (Rio de Janeiro) were randomly selected. In each residence selected, all of the residents aged between 15 and 75 were identified and later chosen using the Kish method. In São Paulo, the three most violent groups were oversampled [16].

The study from the Millennium Institute included a protocol containing 13 instruments [12]. For the present paper, the following instruments were used: (1) sociodemographic questionnaire and (2) Composite International Diagnostic Interview, CIDI 2.1 (OMS), the validated version in Brazil [17, 18].

The psychiatric diagnoses rated in the present study were as follows: (a) alcohol dependence syndrome and (b) Posttraumatic Stress Disorder (PTSD). The rating was done with version 2.1 of the Composite International Diagnostic Interview (CIDI 2.1), lifetime version, which is a structured interview that generates psychiatric diagnoses using the diagnostic criteria of the International Classification of Diseases (ICD-10) and of the Diagnostic Statistical Manual (DSM-IV). The Brazilian version of the CIDI 2.1 was translated and validated by Quintana et al. [17], with sensitivity and specificity for disorders secondary to alcohol use (79.5% e 97.3%). When compared with the Structured Clinical Interview (SCID), the PTSD section of the CIDI 2.1 presented a sensitivity of 82.4% and a specificity of 84.8%, using the criteria from the ICD-10 and sensitivity of 51.5% and specificity of 94.1% for the criteria of the DSM-IV [18]. The diagnoses were generated with a diagnostic algorithm from CIDI 2.1 using the diagnostic criteria from the DSM-IV. The list of traumatic events on the CIDI 2.1 was revised and adapted and 18 new events were added to the original 11 events, adding an evaluation on the intensity of the event, number of times, and ages of the first and last occurrences for each of the 29 events. The standardization, reliability, and validating of the included events were published by Quintana et al. [19]. The events were considered severely traumatic if the interviewee rated the level of intensity of the event as 5 on a scale from 1 to 5.

To group the traumatic events, the authors modified Breslau's classification [20]. The class “assaultive violence” was divided in family violence, sexual crime, spousal violence, criminal violence, and new forms of crime-related violence, which includes the events related to the criminal organization PCC and to being threatened via telephone.

The statistical procedures were executed with Statistical Package for Social Sciences (SPSS, 19th version) for Windows and STATA version 10.0. Due to the multistage, stratified design and the oversampling of the most violent areas, all of the analyses were weighted to control for the effect of the different probabilities of selection in each stage, via a calculation for complex samples done on STATA.

The statistical analyses, both exploratory and inferential, were completed with a sample divided by location of the interview. Through contingency tables, the association between alcohol dependence syndrome and sociodemographic data, severe traumatic events (indicated by a rating of 5), and PSTD was verified. To further evaluate the temporal relationship of the association between alcohol dependence syndrome and traumatic events, variables were generated based on the age of onset of alcohol dependence syndrome and the age of the first and most recent traumatic events.

Severe traumatic events that were found to be significantly associated with alcohol dependence syndrome were grouped based on the temporal relationship between the traumatic event and the onset of the syndrome, respecting the coherence of the nature of the events, resulting in the grouping displayed in Table 1.

From the results described above, two models of logistic regression were elaborated on using alcohol dependence syndrome as the dependent variable. The first model was carried out with the entire sample, including traumatic events and sociodemographic data. The second model included PTSD and was carried out with only those interviewees who suffered a severe traumatic event.

To evaluate the temporal relationship between the occurrence of traumatic events and the establishment of alcohol dependence syndrome, variables considering the age of onset of alcohol dependence syndrome and the age of the first and most recent traumatic event were used to create the groups described above. Finally, another variable was generated, based on the age of onset for alcohol dependence syndrome and the age of the first and most recent traumatic event, grouping all of the traumatic events significantly associated with alcohol dependence syndrome. This variable was compared with sociodemographic data and PTSD through contingency tables and later a logistic regression.

## 3. Results

The final sample included 2,536 interviews in São Paulo and 1,208 in Rio de Janeiro, with corresponding rates of response of 84.5% and 80.5%, respectively.

Among the interviewees in both cities, the predominant gender was female (57.7%, IC = 55.7–59.6) as well as individuals who were married or cohabitating (55.5%, IC = 53.5–57.4). In regard to age, 21.4% (19.8–23.1) of the interviewees were in the third decade of life, and 22.7% (21.0–24.3) were in the fourth decade of life. With regard to education, 39.5% (37.6–41.5) of the interviewees had between nine and twelve years of study. Almost half (44.8%, IC = 42.9–46.8) of the interviewees reported histories of migration, the majority of interviewees (59.2%, IC = 57.2–61.1) were employed, and less than one-third (23.4%, IC = 21.8–25.1) reported a family history of mental illness (Table 2).

In São Paulo, 9.3% (7.1–11.5) of men were diagnosed with alcohol dependence syndrome, and 3.3% (2.2–4.4) of women were diagnosed, totalling 5.8% (4.7–6.9) across genders. In Rio de Janeiro, 8.8% (6.0–11.5) of the men were diagnosed, and 4.2% (2.6–5.9) of the women were diagnosed, totaling 6.2% (4.7–7.7) across both genders. When considering disorders related to alcoholism and the occurrence of alcohol dependence syndrome and/or alcohol abuse, the lifetime prevalence was as follows: in São Paulo, 22.2% (18.3–26.1) in men, 4.9% (3.9–5.9) in women, and 14.2% (12.1–16.3) in both genders; in Rio de Janeiro, 24.3% (19.7–28.9), 9.6% (6.6–12.7), and 17.4% (14.2–20.5), respectively. These prevalence rates were similar in both cities, predominantly in males (*P* = 0.000), except when examining alcoholism in women, which was almost twice as high in Rio de Janeiro compared to São Paulo (*P* = 0.005). However, when considering just alcohol dependence syndrome, there is no significant difference between women in Rio de Janeiro and São Paulo.

In general, 86.4% of interviewees reposted the occurrence of at least one traumatic event in their lifetime. When considering traumatic events self-reported as severe (rated as 5 on a 1–5 scale), 64.9% (62.2–67.3) of interviewees in São Paulo reported a severe traumatic event, 60.8% (57.1–64.4) of men and 67.9% (64.9–71) of women. In Rio de Janeiro, 66.9% (64–69.9) of interviewees reported a severe traumatic event, 61.1% (56.5–65.7) of men and 71.4% (67.6–75.2) of women. There was no significant difference between the two cities (Table 3).

In the logistic regression model carried out in the total sample, including traumatic events and sociodemographic data, alcohol dependence syndrome was associated with males, younger age, less education, having suffered a severe, life-threatening accident, having suffered criminal violence and familiar violence, and having been beaten by a person outside the family in São Paulo. In Rio de Janeiro, alcohol dependence syndrome was associated with males, being single, having suffered criminal violence, having suffered violence associated with new types of crime, and having suffered familial violence. See Table 4.

In the second model, which included PTSD and was carried out with only those interviewees who reported having suffered a severe traumatic event, similar associations were found in both cities, and PTSD was found to be related to alcohol dependence syndrome in São Paulo (*P* = 0.016; Odds = 1.885; IC = 1.128–3.15).

In the analysis of the temporal relationship between the occurrence of traumatic events associated with alcohol dependence syndrome and the onset of this syndrome, it was found that a large portion of traumatic events occurred before the onset of alcohol dependence syndrome in both cities. The exception was found with events grouped as “new types of violence and criminality,” namely, events related to FCC and threats by phone. More than one-fifth of the individuals dependent on alcohol who reported violent and criminal events in both cities suffered violence before and after becoming dependent on alcohol, which characterizes revictimization.

In São Paulo, 20.5% of individuals dependent on alcohol also suffered accidents before and after becoming dependent on alcohol. Adding together all of the traumatic events associated with alcohol dependence syndrome, 55% of the interviewees in São Paulo who were dependent on alcohol also suffered a severe traumatic event, and 68.8% of interviewees in Rio de Janeiro reported severe traumatic events before and after becoming dependent on alcohol, characterizing revictimization. In the city of São Paulo, having PTSD was associated with revictimization (*P* = 0.014; Odds = 3.33) (Tables 5, 6, and 7).

## 4. Discussion

Brazilian studies show an elevated consumption of alcohol, and the age of initial use has been 12.5 years, with lifetime frequency at 65.2% in young students from 12 to 17 years [21]. In a household survey conducted in the Brazilian adult population, Laranjeira and colleagues [22] found that 48% of the sample reported not having drunk alcohol in the past year. However, within those who drank, 29% usually consumed 5 or more drinks per occasion (38% of men). In the total sample, including those who do not drink, 3% met criteria for alcohol abuse, and 9% met criteria for alcohol dependence.

Intoxication, by drugs and more importantly by alcohol [23], was found in 3.1% of interviewees (IC = 1.4–4.8), 5.8% of men (IC = 3.5–8.1), and 1.2% of women (IC = 0.1–2.3), who also have already hurt themselves when intoxicated with alcohol or drugs, and 0.7% of interviewees, of which 1.4% of men (IC = 0.2–2.5) and 0.3% of women have already hurt someone else while intoxicated with drugs or alcohol. Looking at assaultive violence in this same study, 2.3% (IC = 0.8–3.8) of interviewees, of which 4.1% of men (IC = 2.1–6.1) 11 and 1% of women (IC = 0–2.0), have already assaulted someone when intoxicated with alcohol or other drugs; this study did not differentiate between intoxicating substances.

If population-wide studies have demonstrated a strong relationship between the exposure to violence and the development of various mental disorders, including PTSD and disorders related to alcohol use [3], alcohol use as self-medication by people already exposed to tragic events would further expose these people to new traumatic situations, setting the vicious cycle. This chaining of events can occur independently of the emergence of PTSD; alcohol use predisposes trauma that increases alcohol use in the hope of trying to deal with this pain. Repeated exposure to traumatic events has been associated with disorders related to alcohol use, and this association has been more strongly observed in women and adolescents [8, 10, 11].

Brazilian surveys on alcohol misuse consider the prevalence of the abuse and dependence on alcohol, not only on dependence syndrome. Laranjeira and colleagues [22] found a prevalence rate of 19% in men, 4% in women, when considering the Brazilian average. The prevalence found in the present study is similar to the city of São Paulo, 22.2% in men and 4.9% in women. However, in Rio de Janeiro, a larger prevalence of abuse and/or alcohol dependence was found in women, at 9.6%. Even so, this prevalence is still much lower than that of men, which is 24.3%. Coherent with the findings in the present study, all of the national surveys have found prevalence 3 to 4 times greater in men than in women [21, 22, 24]. In a similar form to the present study, Andrade et al. [24] found the association between alcohol dependence syndrome and younger adults and less education.

The finding of the prevalence of exposure to traumatic events throughout the lifespan was high in both cities, 89.5% in Rio de Janeiro and 86% in São Paulo. Compared to other studies in Brazil and in other Latin American countries, the general levels of exposure to traumatic events were higher in the present study, even when considering only the events self-reported as severe, with an intensity level of 5 [24–26]. Differences like these can be explained by a broader list of traumatic events used in the present study. When comparing the same traumatic events, Brazilians are discretely more exposed to urban violence, as domestic and sexual violence is more prevalent in Mexico [25] and in Chile [26]. Compared to the literature, the present study found lower rates of domestic violence. This difference is most likely due to the method, as many of the previous studies were designed to measure violence [12, 27–31] in specific populations, such as children and adolescents [27–29] and women [30, 31], using more comprehensive instruments.

The associations found between alcohol dependence syndrome and traumatic events, including violence and criminality, are strongly established in the literature, as was demonstrated in the Introduction [4, 32]. The mediation of this association by PTSD is also well studied [3, 7]. Breslau's study [7] found that the development of alcoholism is related to PTSD but not to the occurrence of traumatic events without the development of this mental disorder. In the present study, an association was found between PTSD and alcohol dependence syndrome in São Paulo and not Rio de Janeiro. Similar to the larger prevalence of alcohol abuse in women in Rio de Janeiro, cultural differences can collaborate to get these results. This divergence shows the importance of moderation when comparing data from studies from one population to another, reinforcing the importance of the production of regional data.

When conducting the logistic regression, sexual and conjugal violence did not have a significant relationship to alcohol dependence syndrome. With regard to sexual violence, a possible explanation for this result is the low prevalence of these types of events reported by men: 0.2% in São Paulo and 1.3% in Rio de Janeiro, probably due to underreporting of these events in this sample. The literature reports this difficulty, due to the feelings of guilt and shame of the victim, especially strong in today's male population [33]. Since alcohol dependence syndrome is more prevalent in men, underreporting may have skewed these results.

Similarly, the prevalence rates found of conjugal violence suffered by men are equally low: 0.4% in São Paulo and 1% in Rio de Janeiro. Here, the reason for this finding is most likely the method. In a study examining dynamics of violence in couples, Carvalho et al. [34] propose that, regardless of prevalence, female aggression against men occurs with less severity of violence. When considering only physical violence of great intensity, this occurs basically against women, presenting lower prevalence in alcohol dependence syndrome. Studies that compare alcohol use and domestic violence do not discuss alcohol dependence exclusively [4, 21]. Acute alcohol intoxication, which often is used to measure this category of violence, was not measured in the present study.

As was presented in the above introduction, traumatic events of diverse types and Posttraumatic Stress Disorder are complexly related with the existence or development of alcohol dependence syndrome. An important aspect of this complexity is the temporal relationships between the events. Despite being a cross-sectional, epidemiological study, when considering the age at which traumatic events occurred and the development of alcohol dependence syndrome, this discussion can be advanced. Among the individuals dependent on alcohol who have suffered severe accidents, 61.4% in São Paulo and 55.6% in Rio de Janeiro suffered accidents in the onset of alcohol dependence. Among the individuals dependent on alcohol who reported violent and criminal events, 45.5% in São Paulo and 50.9% in Rio de Janeiro suffered traumatic events before the development of alcohol dependence. The exception is found in the cases of threats by telephone and attacks by PCC (the wave of violence against security forces and some civil targets organized by the criminal organization “First Capital Command” (PCC), originating in the city of São Paulo on May 12, 2006, which subsequently spread through the state of São Paulo and other states in Brazil. For two days, the city of São Paulo became immersed in an intense climate of terror which caused the closing of commercial establishments and the emptying of the streets even during business hours); in Rio, 92.6% of individuals were already dependent on alcohol by the time of the violent events. This is possibly due, because they were recent events, to not having enough time to develop alcohol dependence syndrome. Alcohol intoxication, independent of alcohol dependence syndrome, predisposes an individual to accidents of various types [2], as it can facilitate the occurrence of violent events related to criminality, like theft, assaults, and kidnapping [2]. While being intoxicated by alcohol, the individual's vigilance, reflexes, and capacity for reaction are reduced, becoming more vulnerable. Alcohol dependence syndrome is insidious, taking years to be established, and is often preceded by improper use of alcohol [2]. In this way, even if the alcohol dependence has been previously established, this does not mean that alcohol use was not previously related to violent accidents and events, but this cannot be measured in the present study. On the other hand, the literature corroborates the present finding that traumatic experiences of a violent accident or situation lead to the onset of alcohol dependence syndrome, especially when mediated by PTSD [35]. This hypothesis is based on the notion that alcohol use is a form of self-medication, observed in clinical populations [36]. Some authors have observed that a decrease in PTSD symptoms would concomitantly cause a decrease or improvement in alcohol dependence [37]. In a study conducted with women who have suffered violence, Kaysen et al. [38] found that the intensity with which alcohol was ingested proportionally increased the intensity of traumatic symptoms, reinforcing the thesis of self-medication.

“Taking a beating” from someone who is not a member of the family was reported by 2.4% of the interviewees in São Paulo and 2.6% of the interviewees in Rio de Janeiro, with a traumatic experience rated at the most severe intensity level. This event is associated with alcohol dependence syndrome in São Paulo and preceded the syndrome in 93.8% of cases in São Paulo and 85.7% in Rio de Janeiro. There were no cases of recidivism (a traumatic event before and after the alcohol dependence) in either city. According to common sense, an individual dependent on alcohol who reports a history of being beaten by a stranger is often interpreted as someone who involved themselves in a fight while intoxicated. The data does not corroborate this hypothesis, because this type of event does not repeat itself after the onset of the alcohol dependence. In this case, the self-medication hypothesis, or the increase in alcohol use to deal with symptoms resulting from a traumatic experience, is again more strongly suggested [35]. The prevalence rate of interviewees who reported physical violence perpetrated by a family member is 11.5% in São Paulo and 14.5% in Rio de Janeiro. In more than 90% of cases, the violence preceded the alcohol dependence. This temporal relationship is expected, because this form of violence generally occurs during infancy. In the Bordin et al. study [27], experiencing severe physical punishment was associated with emotional problems and behavioural issues in children and adolescents. Paula et al. [28] showed that adolescents exposed to violence within the family had twice as many problems with mental health as those who were not exposed to intrafamiliar violence and were three times more likely to present with mental health issues than those exposed to only urban violence (*P* = 0.04; IC 95% = 1.03; 7.55). The explanation for this relationship between violence suffered during infancy and the development of mental disorders involves many factors, among which is the alteration of reactions to stress, measured by the hypothalamus-pituitary-adrenal axis (HPA). Studies with animal models and brain imaging studies in adults who have suffered severe traumas during early childhood show that early trauma can determine alterations of the HPA axis or structural modifications of the nervous system, with permanent damage [39].

Other factors associated with the examination of childhood domestic violence are sociocultural. In a national survey examining the consumption of psychiatric drugs by elementary and secondary school students carried out in the public education system in the 27 Brazilian state capitals [5], the negative parent-child relationship of the students and negative relationships between parents of the students were found to be associated with heavy alcohol use by students, and the moderation by the parents in the treatment of their children was found to be associated with students not using alcohol heavily.

Other studies show the relationship between having suffered violence during childhood or witnessing domestic violence during childhood and violent behaviour during adulthood [40]. Braga [41] explains that parents as well as social environment have a direct impact on children, who are learning cultural norms of behaviour. In other words, an individual raised with corporal and violent punishments can comprehend these practices as a form of education and not as violence. Here, the vicious cycle of violence, alcohol, and violence perpetuates itself over generations.

The relationship between suffering violence and development of alcohol dependence may not be causal but may be due to underlying factors. Epidemiological studies show that an individual who lives in violence finds himself more vulnerable to mental illness and especially the development of alcoholism [24]. The phenomenon of the banality of violence, the idea that a culture of violence exists in certain environments, does not diminish the damaging effects of this violent behaviour. On the contrary, chronic exposure to trauma, whether continuous or intermittent, is capable of altering the HPA axis, leading to functional losses in the body and mental illness [42]. This pathological modulation of the HPA axis can predispose PTSD, which in turn predisposes alcohol dependence syndrome and new traumatic events [7, 39].

On the other hand, the literature shows the influence of alcohol use on the genesis of violent behaviour [23]. The question of the vicious cycle returns, which is demonstrated in the present study. Adding together all of the severe traumatic events related with alcohol dependence, 55% of individuals dependent on alcohol in São Paulo and 68.8% in Rio de Janeiro report events occurring both before and after the development of alcohol dependence. Suffering trauma predisposes alcohol dependence, which in turn predisposes new traumas and violent events, in the same individual and also within families, for generations, collaborating with the perpetuation of a violent society.

Finally, various discrepancies were found in the results of these two cities, within the relationships of the prevalence rates as well as the correlations of alcohol dependence syndrome. These discrepancies were minimized with the creations of the groups. Cultural and sociological factors possibly implicated in these differences fall out of the scope of this project.

New studies with other designs, including a qualitative approach, are necessary to clarify these points. Longitudinal studies as well as regional studies are also necessary to enlarge and deepen the comprehension of these findings.

## 5. Conclusion

Alcohol dependence syndrome is complexly related to urban and familiar violence in the cities of São Paulo and Rio de Janeiro. Urban violence, in more than 50% of cases, and family violence, in more than 90% of cases, precede alcohol dependence syndrome.

In both cities, the reoccurrence of traumatic events occurs in more than half of individuals with alcohol dependence, characterizing a vicious cycle that contributes to the perpetuation of high rates of alcoholism as well as a violence society. Politicians ordering the reduction of violence in large metropolises are necessary to break this cycle, potentially reducing, as a result, alcoholism in this population.

## Figures and Tables

**Table 1:** Grouping of traumatic events related to alcohol dependence syndrome.

(1) Accidents	
(a) Suffering a life-threatening car or motorcycle accident	
(b) Suffering another type of life-threatening accident	
(2) Violence and criminality	
(a) Physically assaulted or attacked without weapon	
(b) Surrendered, assaulted, or threatened with a weapon	
(c) Maintained in captivity or kidnapped	
(d) Tortured or being victim of terrorism	
(e) Threatened with death	
(f) Being victim of gang wars or drug traffickers	
(g) Being home when an intruder attempted to invade your home	
(h) Witnessing someone suffer a severe, life-threatening injury or witnessing someone being killed	
(i) Witnessing gunfire	
(j) An intruder attempted to invade your home when you were not at home	
(k) Seeing a corpse, except at funerals, or had to touch a corpse for any reason	
(l) Seeing atrocities or carnages, such as mutilated corpses or killings	
(3) New forms of crime-related violence	
(a) Witnessing or suffering consequences from attacks of the First Capital Command, PCC^1,2^;	
(b) Becoming stressed with the attacks of PCC	
(c) Being threatened via telephone	
(4) Sexual crime	
(a) Rape, which is when someone has had a sexual relationship with you that you did not want or threatened you or used force	
(b) Being abused sexually, which is when someone touches you or feels your genitals when you did not want this	
(5) Family violence	
(a) A family member beat you with enough force to cause injuries	
(b) Witnessing during childhood a severe fight with physical aggression at home	
(6) Illness or death of a person close to you	
(a) Nonviolent sudden death of a person close to you	
(b) Relative or close friend had a life-threatening illness or injury	
(7) Spousal violence: physically assaulted by a spouse	
(8) Getting beaten by someone else (except spouse or family)	

^1^This question and the following question refer to the wave of violence against security forces and some civil targets organized by the criminal organization “First Capital Command” (PCC), originating in the city of São Paulo on May 12, 2006, which subsequently spread through the state of São Paulo and other states in Brazil. For two days, the city of São Paulo became immersed in an intense climate of terror which caused the closing of commercial establishments and the emptying of the streets even during business hours.

^2^For interviews conducted in Rio de Janeiro, the question was modified to include an active criminal organization equivalent to PCC that, during the sequence of events in São Paulo, perpetrated violent acts against the population, such as assaults on mass transit, followed by lighting a bus on fire with passengers aboard.

**Table 2:** Sample distribution sociodemographic characteristics (*n* = 3,744).

	São Paulo	Rio de Janeiro	Total
	% (95% IC)	% (95% IC)	% (95% IC)
Gender			
Masculine	41.9 (39.5–44.3)	43.4 (40.3–46.5)	42.3 (40.4–44.3)
Feminine	58.1 (55.7–60.5)	56.6 (53.5–59.7)	57.7 (55.7–59.6)
Age (years)			
15–19	8.5 (7.2–9.7)	8.4 (6.7–10.1)	8.4 (7.4–9.5)
20–29	22.9 (20.8–24.9)	17.9 (15.5–20.2)	21.4 (19.8–23.1)
30–39	23.9 (21.8–25.9)	19.6 (17.2–22.0)	22.7 (21.0–24.3)
40–49	17.1 (15.3–19.0)	19.9 (17.4–22.4)	17.9 (16.4–19.4)
50–59	15.7 (13.8–17.5)	16.4 (14.1–18.7)	15.9 (14.4–17.4)
60–69	8.1 (6.8–9.5)	11.6 (9.6–13.6)	9.1 (8.0–10.3)
70–75	3.9 (2.9–4.9)	6.3 (4.7–7.8)	4.6 (3.7–5.4)
Average (standard deviation)	39.5 (38.7–40.3)	42.4 (41.3–43.4)	40.3 (39.7–40.9)
Marital status			
Single	28.3 (26.1–30.5)	31.4 (28.4–34.3)	29.2 (27.4–31.0)
Married/cohabitating	56.9 (54.5–59.3)	51.9 (48.9–55.0)	55.5 (53.5–57.4)
Separated/divorced	9.0 (7.6–10.4)	10.7 (8.8–12.7)	9.5 (8.3–10.6)
Widowed	5.8 (4.6–7.0)	6.0 (4.5–7.5)	5.9 (4.9–6.8)
Education			
0 to 4 years	20.0 (18.1–21.9)	14.6 (12.4–16.8)	18.5 (17.0–20.0)
5 to 8 years	25.5 (23.4–27.6)	23.1 (20.4–25.7)	24.8 (23.2–26.5)
9 to 12 years	38.9 (36.5–41.3)	41.1 (38.1–44.2)	39.5 (37.6–41.5)
13 years and older	15.6 (13.7–17.5)	21.2 (18.7–23.7)	17.2 (15.6–18.7)
Employment status			
Currently employed	60.3 (57.3–62.7)	56.4 (53.3–59.6)	59.2 (57.2–61.1)
With income	59.0 (56.5–61.5)	55.5 (52.0–58.3)	57.9 (55.9–59.9)
Migration history			
Migrant	50.0 (47.6–52.5)	31.7 (28.8–34.6)	44.8 (42.9–46.8)
Family history of psychiatric disorder			
Yes	22.1 (20.1–24.2)	26.7 (23.9–29.4)	23.4 (21.8–25.1)

**Table 3:** Prevalence of traumatic events (*n* = 3,744).

	São Paulo			Rio de Janeiro		
	Males	Females	Total	Males	Females	Total
	% (95% IC)	% (95% IC)	% (95% IC)	% (95% IC)	% (95% IC)	% (95% IC)
Accidents	16.06 (13.28–18.85)	11.98 (9.87–14.09)	13.69 (11.99–15.39)	15.92 (12.49–19.36)	14.11 (11.16–17.06)	14.90 (12.66–17.14)
Criminal violence	46.18 (42.44–49.92)	44.85 (41.62–48.09)	45.41 (42.96–47.85)	47.45 (42.71–52.19)	52.62 (48.45–56.79)	50.38 (47.24–53.51)
New forms of criminal violence	9.15 (6.94–11.37)	18.99 (16.42–21.57)	14.87 (13.09–16.65)	12.29 (9.25–15.34)	22.14 (18.66–25.63)	17.87 (15.47–20.27)
Sexual violence	0.20 (0.00–0.39)	4.01 (2.83–5.18)	2.41 (1.72–3.10)	1.31 (0.24–2.38)	6.79 (4.68–8.90)	4.41 (3.12–5.70)
Family violence	6.79 (4.90–8.67)	14.91 (12.68–17.14)	11.51 (9.98–13.03)	10.00 (7.08–12.91)	17.88 (14.71–21.05)	14.46 (12.26–16.67)
Death or severe illness of relative or close friend	29.62 (26.16–33.08)	39.44 (36.27–42.62)	35.33 (32.97–37.68)	33.20 (28.68–37.73)	42.65 (38.51–46.78)	38.55 (35.48–41.62)
Getting beaten by a person (except relatives or torture)	1.57 (0.64–2.49)	2.96 (1.80–4.12)	2.37 (1.60–3.15)	3.10 (1.64–4.57)	2.21 (1.11–3.32)	2.60 (1.71–3.49)
Spousal violence	0.44 (0.15–0.73)	6.57 (5.03–8.11)	4.00 (3.09–4.91)	1.01 (−0.09–2.10)	7.30 (5.18–9.41)	4.57 (3.27–5.87)

**Table 4:** Logistic regression conducted by interview location, including traumatic events and sociodemographic data (SP = 2,536; RJ = 1,208).

Location		*P*	Odds ratio	IC (95%)	
				Minimum	Maximum
São Paulo	Male	3.67	0.00	2.42	5.58
	Age (years)	0.98	0.01	0.96	1.00
	Education (years)	0.93	0.00	0.89	0.97
	Civil state				
	Married	Reference			
	Widowed	0.55	0.36	0.15	1.98
	Separated/divorced	1.62	0.14	0.85	3.06
	Single	0.92	0.70	0.59	1.43
	Traumatic event				
	No	Reference			
	Severe accident	1.92	0.00	1.25	2.95
	Violence and criminality	1.78	0.01	1.18	2.67
	New forms of criminal violence	1.38	0.24	0.80	2.36
	Sexual violence	2.02	0.09	0.88	4.60
	Family violence	1.86	0.02	1.13	3.09
	Death of a close friend	1.18	0.39	0.81	1.70
	Spousal violence	1.92	0.09	0.90	4.08
	Being beaten by another person (except torture)	4.93	0.00	2.45	9.93

Rio de Janeiro	Male	3.25	0.00	1.90	5.56
	Age (years)	1.01	0.36	0.99	1.02
	Education (years)	0.98	0.47	0.91	1.04
	Civil state				
	Married	Reference			
	Widowed	2.04	0.19	0.70	5.99
	Separated/divorced	1.51	0.31	0.67	3.38
	Single	2.43	0.00	1.37	4.30
	Traumatic event				
	No	Reference			
	Severe accident	1.10	0.74	0.63	1.91
	Violence and criminality	2.23	0.01	1.17	4.25
	New forms of criminal violence	2.18	0.00	1.29	3.66
	Sexual violence	1.85	0.16	0.78	4.38
	Family violence	2.93	0.01	1.30	6.60
	Death of a close friend	1.31	0.24	0.83	2.05
	Spousal violence	1.02	0.97	0.34	3.04
	Being beaten by another person (except torture)	1.26	0.59	0.54	2.96

**Table 5:** Proportion of individuals who suffered traumatic events related to alcohol before, after, or before and after the onset of alcohol dependence syndrome (SP = 2,536; RJ = 1,208).

	São Paulo		Rio de Janeiro	
	*N*	%	*N*	%
“Accident after alcohol”	8	18.2	7	38.9
“Accident before alcohol”	27	61.4	10	55.6
Accident before and after alcohol	9	20.5	1	5.6
Total	**44**	**100**	**18**	**100**

“Violence and criminality after alcohol”	26	26.3	12	22.6
“Violence and criminality before alcohol”	45	45.5	27	50.9
Violence and criminality before and after alcohol	28	28.3	14	26.4
Total	**99**	**100**	**53**	**100**

“New violence after alcohol”	28	84.8	25	92.6
“New violence before alcohol”	5	15.2	2	7.4
Total	**33**	**100**	**27**	**100**

“Familiar violence after alcohol”	1	2.6	1	4.2
“Familiar violence before alcohol”	36	94.7	22	91.7
Familiar violence before and after alcohol	1	2.6	1	4.2
Total	**38**	**100**	**24**	**100**

Alcohol dependence syndrome before being beaten by strangers	1	6.2	1	14.3
Being beaten by strangers before alcohol dependence syndrome	15	93.8	6	85.7
Total	**16**	**100**	**7**	**100**

**Table 6:** Proportion of individuals with a traumatic event related to alcohol and dependence syndrome, including proportion of individuals who suffered events before, after, or before and after the onset of alcohol dependence syndrome (SP = 2,536; RJ = 1,208).

Location		*N*	%	% of individuals with trauma and alcohol dependence syndrome
São Paulo	No severe traumatic event and no alcohol	845	33.4	
	Severe traumatic event and no alcohol	1542	60.9	
	Trauma and alcohol			
	“Trauma after alcohol”	19	0.8	15.8
	“Trauma before alcohol”	35	1.4	29.2
	Trauma before and after alcohol	66	2.6	55.0
	Total trauma + Alcohol dependence syndrome	120	4.8	100.0
	Alcohol and no traumatic event	26	1	
	Total	**2533**	**100**	

Rio de Janeiro	No severe traumatic event and no alcohol	374	31	
	Severe traumatic event and no alcohol	760	63	
	Trauma and alcohol			
	“Trauma after alcohol”	12	1	18.8
	“Trauma before alcohol”	8	0.7	12.5
	Trauma before and after alcohol	44	3.6	68.8
	Total trauma + alcohol dependence syndrome	64	5.3	100.0
	Alcohol and no severe traumatic event	8	0.7	
	Total	**1206**	**100**	

**Table 7:** Logistic regression conducted with only those who suffered serious traumatic events and also presented with a diagnosis of alcohol dependence syndrome, by location of interview, including PTSD and sociodemographic data compared with trauma suffered before and after alcohol dependence syndrome.

Location of interview		*P*	Odds ratio	IC (95.0%)	
				Minimum	Maximum
São Paulo	Male	0.68	0.84	0.36	1.94
	Age (years)	0.20	1.02	0.99	1.05
	Education (years)	0.88	1.01	0.91	1.12
	Civil state				
	Married	Reference			
	Widowed	0.50	0.35	0.02	7.18
	Separated/divorced	0.39	0.62	0.21	1.86
	Single	0.53	1.36	0.52	3.53
	PTSD	0.01	3.33	1.28	8.67

Rio de Janeiro	Masculine	0.20	2.17	0.65	7.22
	Age (years)	0.65	0.99	0.94	1.04
	Education (years)	0.88	0.99	0.85	1.14
	Civil state				
	Married	Reference			
	Widowed	0.04	0.05	0.00	0.88
	Separated/divorced	0.54	2.10	0.19	23.14
	Single	0.13	0.34	0.08	1.39
	PTSD	0.27	2.36	0.51	10.84
